# Study on Structural Design and Motion Characteristics of Magnetic Helical Soft Microrobots with Drug-Carrying Function

**DOI:** 10.3390/mi15060731

**Published:** 2024-05-31

**Authors:** Qian Gao, Tingting Lin, Ziteng Liu, Zebiao Chen, Zidong Chen, Cheng Hu, Teng Shen

**Affiliations:** 1 Luohe Institute of Technology, Henan University of Technology, No. 123, University Road, Yuanhui District, Luohe 462000, China; higaoqian@163.com; 2 Higher Education Mega Center, Guangzhou University, No. 230, West Waihuan Street, Guangzhou 510006, China; 2112307013@e.gzhu.edu.cn (T.L.); 2112207121@e.gzhu.edu.cn (Z.L.); 2112207061@e.gzhu.edu.cn (Z.C.); 2112307103@e.gzhu.edu.cn (Z.C.)

**Keywords:** magnetic soft microrobots, magnetic actuation, helical propulsion, structure preparation, motion characteristics

## Abstract

Magnetic soft microrobots have a wide range of applications in targeted drug therapy, cell manipulation, and other aspects. Currently, the research on magnetic soft microrobots is still in the exploratory stage, and most of the research focuses on a single helical structure, which has limited space to perform drug-carrying tasks efficiently and cannot satisfy specific medical goals in terms of propulsion speed. Therefore, balancing the motion speed and drug-carrying performance is a current challenge to overcome. In this paper, a magnetically controlled cone-helix soft microrobot structure with a drug-carrying function is proposed, its helical propulsion mechanism is deduced, a dynamical model is constructed, and the microrobot structure is prepared using femtosecond laser two-photon polymerization three-dimensional printing technology for magnetic drive control experiments. The results show that under the premise of ensuring sufficient drug-carrying space, the microrobot structure proposed in this paper can realize helical propulsion quickly and stably, and the speed of motion increases with increases in the frequency of the rotating magnetic field. The microrobot with a larger cavity diameter and a larger helical pitch exhibits faster rotary advancement speed, while the microrobot with a smaller helical height and a smaller helical cone angle outperforms other structures with the same feature sizes. The microrobot with a cone angle of 0.2 rad, a helical pitch of 100 µm, a helical height of 220 µm, and a cavity diameter of 80 µm achieves a maximum longitudinal motion speed of 390 µm/s.

## 1. Introduction

With their advantages of small size, light weight, and flexible movement, soft nano- and microrobots are expected to become a new generation of key technologies applied in targeted drug delivery, non-invasive surgery, cell transplantation, and other life and health fields [1,2,3,4,5,6,7]. Due to their structural scale characteristics, soft nano- and microrobots cannot be powered by built-in drives like macro robots, and need to utilize external drives to realize energy supply. Therefore, microrobots with different driving principles have been rapidly developed in the past two decades, and a wide range of chemical and external physical field (optical, electric, magnetic, thermal, etc.)-driven swimmable robots have gradually emerged [8,9,10,11]. In terms of comprehensive analysis, for complex microvascular environments, chemical actuation is a complex and poorly controllable process, while electric field actuation cannot be used directly in living organisms; although optical field actuation has the potential for targeted in vivo internal medical treatment, it requires a certain degree of transparency and has a weak penetration ability; ultrasonic field actuation has a high degree of penetration into living organisms, but it has low control precision [12,13,14,15,16,17]. In contrast, magnetic drive has the characteristics of non-contact, low frequency, and high control precision, and it is harmless to living organisms, so it has received extensive attention from scholars at home and abroad [18]. 

Based on the magnetic drive mechanism, different types of magnetic vascular microrobots have been introduced one after another by imitating the motions of microorganisms in nature, which can be mainly categorized into three types: magnetic force-driven motion, magnetic force-driven oscillating motion, and magnetic moment-driven spiral motion [19,20,21]. Among many magnetic microrobots inspired by the spiral propulsion behavior of microorganisms, the micro-helix robot is undoubtedly the best performer, as it not only has the characteristics of flexible spatiotemporal manipulation, strong propulsion, and good biocompatibility, but it also utilizes the helical propulsion method, which is more conducive to penetrating the tissue of the diseased area [22,23]. For example, in 2009, Li Zhang et al. from ETH Zurich proposed a helical microrobot controllable in liquid, consisting of a soft magnetic metal head in the shape of a square sheet and a helical nanoribbon tail resembling a natural flagellum [22]. A uniform rotating magnetic field was generated by three orthogonal electromagnetic coils in order to achieve precise control with a maximum speed of 18 µm/s. In the same year, Ambarish Ghosh et al. from Harvard University used the glancing angle deposition (GLAD) technique to prepare a nanoscale helical microrobot with a diameter of 200 nm, a body length of 2 µm, and a cobalt-covered surface, which could be rotated in solution to advance 212 nm per rotation [24]. In 2017, Zhang Li’s group prepared a magnetic microrobot with superparamagnetic properties by dipping helical micro-algae into magnetite (Fe_3_O_4_) suspension, resulting in a robot with good movement and navigation capabilities in a variety of biological fluids [23]. In 2018, Antoine et al. investigated the kinematic modes of a helical microrobot with conical head and explored the targeting manipulation methods in different pipelines [25]. In 2019, Chen et al. proposed a magnetic microconical helical robot structure, and investigated the target manipulation and drug release properties of this robot in static fluids [26]. In 2021, Lee et al. prepared a biodegradable magnetically controlled helical soft microrobot by two-photon polymerization laser direct-write technology, and investigated its animal tissue fluids with offset characteristics and out-of-step frequency characteristics [27]. And in 2023, Park et al. investigated a porous degradable magnetically controlled helical microrobot that could move up to 12 mm/s in a simple liquid environment, but the angle of offset from the target increased as the speed increased [28].

As can be seen through the above analysis, some progress has been made in the research of magnetic soft microrobots, but most of the research still focuses on the simple helix structure, which can ensure a faster propulsion speed; but the limited space for drug-carrying and the single execution function cannot satisfy the needs of drug delivery and minimally invasive medical treatment [24,29]. Therefore, in order to realize the functional unification of motion performance and drug-carrying performance, this paper proposes a magnetically controlled cone-helix soft microrobot structure, which consists of a conical helix part with an elliptical cross-section and a drug-carrying cavity part. This structure is expected to achieve higher motion speeds and perform efficient drug-carrying work compared to conventional straight helix microrobots with circular cross sections. On this basis, the motion mechanism analysis, dynamics modeling, femtosecond laser 3D printing preparation, and magnetic drive control experiments of the magnetically controlled microrobot were carried out. The drug-carrying cavity of the microrobot brings a wide range of potential applications. The drug-carrying cavity can be functionally expanded as needed, and by loading different types of drugs, targeted drug delivery can be realized for a variety of diseases. If the robot is loaded with nanosensors, the concentrations and release rates of drugs can be monitored in real time to improve therapeutic effects and minimize side effects [30]. When the control technology is mature, by utilizing group intelligence algorithms, multiple microrobots can work together to accomplish complex tasks, such as large-area drug delivery.

## 2. Structure Design and Motion Mechanism

**Figure 1:**
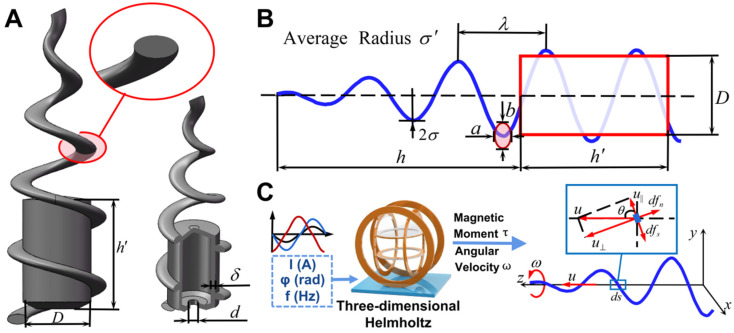
(**A**) 3D structure of cone-helix soft microrobot with elliptical cross section. (**B**) Definition of geometric parameters of cone-helix soft microrobot. (**C**) Schematic representation of forces on arc segment in low-Reynolds number fluid environment.

According to the analysis on the cone-helix structure by Xin Chen of the University of Science and Technology of China [31], the larger the cone angle of the cone-helix microrobot, the larger the declination angle of its magnetic axis, the easier it is for the microrobot structure to realize rotational motion along its short axis in the magnetic field, and the higher the propulsive speed under the condition of the same magnetic field frequency. Accompanied by the longitudinal propulsion motion of the magnetically controlled microrobot, the microrobot will often sink to the bottom, and the bottom surface has a certain friction, thus generating lateral motion; when the size of the cone angle exceeds a certain range, its lateral and longitudinal speeds will be elevated. According to the analysis of the out-of-step frequencies of different average radii in the cone-helix microrobots, the larger the average radius of the cone-helix portion of the microrobot, the smaller the corresponding out-of-step frequency, and potentially, the smaller the speed the microrobot can achieve. In addition, the larger the pitch of the conical helix, the faster the microrobot moves, but its stall frequency also decreases. Among the various helical microrobot structures, helical microrobots with an elliptical cross-section are structurally robust enough to undergo flow and surface tension during development and drying, as well as being able to remain stable during subsequent electron beam evaporation and sputtering. Furthermore, helical microrobots with an elliptical cross-section have a much faster speed relative to the conventional helical microrobot structures with a circular cross-section [21,32]. Considering the influence of structural characteristics on the speed of movement, this paper proposes a new type of micro–nano soft body robot, as shown in Figure 1A—the head of the elliptical cross-section of the conical helical structure connects the tail to a cylindrical cavity, so that the structure can have a certain amount of drug-carrying space, the volume of which accounts for 43.15% of the overall volume of the structure. In order to increase the efficiency of the rotary drive to move forward, the helical structure extends around the drug-carrying cavity.

The motion of a microrobot can be regarded as a swimming problem in a low-Reynolds number fluid environment. At this point, the viscous forces of the fluid dominate over the inertial forces. Due to the conservation of mass in an incompressible Newtonian fluid ∇⋅v=0, the Navier–Stokes equations used to describe the motion of a viscous fluid can be simplified as:

(1) $$\{\begin{array}{c} \nabla p=\eta \nabla^{2}\vec{v}\\ \nabla \cdot v=0 \end{array}$$

where $\vec{v}$ is the flow velocity of the fluid, p is the fluid pressure, and $\eta \nabla^{2}\vec{v}$ is the fluid viscous force. From the above equation, the fluid pressure is linearly related to its local velocity and time has no effect on the motion of the object, i.e., the object moves only in the state of force, and the object in this environment moves with a peristaltic effect.

A magnetically controlled micro–nano soft robot is essentially a kind of microstructure with magnetism, which is also a kind of magnetic medium; magnetization occurs under the action of an external magnetic field, which can make an object show magnetism itself and produce a magnetic field. The magnetically controlled micro–nano soft robot can be regarded as a magnetic dipole after magnetization due to its extremely small size. Denoting the magnetic dipole moment of the microrobot by $\vec{m}$, the potential energy of the microrobot in the external magnetic field, $\vec{B}$, can be expressed as:

(2) $$U=-\vec{m}\cdot \vec{B}.$$

According to the law of conservation of energy, the magnetic force on the microrobot, i.e., a magnetic dipole, in the external magnetic field can be obtained with:

(3) $$F=-\nabla U=\vec{m}\times (\nabla \times \vec{B})+(\vec{m}\cdot \nabla )\vec{B}.$$

Due to the tiny space where the magnetic dipole is located, the calculated region can be regarded as having no external current. According to Maxwell’s equations, the quasi-static magnetic field can be expressed as $\nabla \times \vec{B}=0$, which can be substituted into Equation (3) to obtain:

(4) $$F=(\vec{m}\cdot \nabla )\vec{B}=\nabla \vec{B}\cdot \vec{m}.$$

From the expression of the applied magnetic force, it can be seen that the microrobot will be subjected to the magnetic field force in the presence of a gradient in the external magnetic field, thus causing translational motion. The magnitude of the magnetic moment applied to the microrobot in the external magnetic field is:

(5) $$L=-\frac{\partial }{\partial \theta }U=\frac{\partial }{\partial \theta }mB\cos\theta =-mB\sin\theta$$

where θ is the angle between the direction of the magnetic moment of the microrobot and the direction of the magnetic field. Taking into account the direction of the magnetic moment gives:

(6) $$\vec{L}=\vec{m}\times \vec{B}=\left[\begin{array}{ccc} 0 & B_{z} & -B_{y}\\ -B_{z} & 0 & B_{x}\\ B_{y} & -B_{x} & 0 \end{array}\right].$$

From Equations (5) and (6), it can be seen that when the direction of the magnetic moment of the microrobot is perpendicular to the direction of the magnetic field, it is subjected to the largest magnetic moment, while when the direction of the magnetic moment is parallel to the direction of the magnetic field, the microrobot is subjected to zero magnetic moment. Regardless of a uniform or non-uniform magnetic field, as long as the direction of the magnetic field forms a certain angle with the direction of the magnetic moment of the microrobot, the microrobot will be affected by the external magnetic field and rotate under the action of the magnetic moment. For a microrobot propelled by rotation, we want it to avoid the magnetic field force as much as possible, and only be affected by the magnetic moment, so that it can achieve the purpose of advancing by rotating around its long axis. Therefore, a magnetic field environment with uniform rotation is created by adjusting the current of the electromagnetic coil to change the size and direction of the magnetic field. Driven by this magnetic field environment, the helically propelled micro–nano soft robot is only subjected to the magnetic moment, and its calculation formula can be expressed as follows:

(7) $$\vec{L}=V_{m}\vec{M}\times \vec{B}$$

where $V_{m}$ is the volume of the magnet (m^3^), $\vec{M}$ is the magnetization intensity (A/m), and $\vec{B}$ is the magnetic induction intensity (T).

For the microrobotic structure proposed in this paper, which mainly relies on the helical section at the front to achieve propulsion, its motion in a low-Reynolds number environment is dynamically analyzed. According to the drag theory model, the helix is micronized; Figure 1C shows the force on a certain arc segment, and the velocity $\vec{u}$ of the arc segment ds is decomposed into the velocity components parallel and perpendicular to the arc segment:

(8) $$\left\{ \begin{array}{l} df_{s}=-\zeta_{\left|\right|}u_{\left|\right|}ds\\ df_{n}=-\zeta_{⊥}u_{⊥}ds \end{array}\right.$$

where $u_{\left|\right|}$ is the tangential velocity of the arc segment, $u_{⊥}$ is the normal velocity of the arc segment, $\zeta_{\left|\right|}$ is the tangential drag coefficient, $\zeta_{⊥}$ is the normal drag coefficient, $df_{s}$ is the tangential drag of the arc segment, and $df_{n}$ is the normal drag of the arc segment.

When the microrobot rotates forward with an angular velocity of ω, the linear velocity of each arc segment of the helical section can be expressed as $u_{\theta }=\omega \sigma$, which corresponds to a tangential velocity of $u_{\left|\right|}=u_{\theta }\sin\theta =\omega \sigma \sin\theta$ and a normal velocity of $u_{⊥}=u_{\theta }\cos\theta =\omega \sigma \cos\theta$.

For the conical helix micro–nano soft robot proposed in this paper, the tail of the helix part has a radius different from that of the head, and the linear velocity of the tail is faster than that of the head at a certain rotational frequency. Therefore, the head of the microrobot moves in the opposite direction of rotation to correct a portion of the lateral movement [31].

Purcell proved in his paper that the non-hydrodynamic force F and the non-hydrodynamic torque τ of a helically propelled microrobot along its axis are linearly related to the microrobot’s forward velocity u and rotational angular velocity ω, and can be described by a symmetric matrix [33], denoted as:

(9) $$\left[\begin{array}{c} F\\ \tau \end{array}\right]=\left[\begin{array}{cc} A & B\\ B & C \end{array}\right]\left[\begin{array}{c} u\\ \omega \end{array}\right]$$

where parameters *A*, *B*, and *C* relate to the geometrical and environmental properties of the helical section. The parameters in the symmetry matrix can be expressed as:

(10) $$A=2\pi n\sigma^{\prime }\left(\frac{\zeta_{\left|\right|}\cos^{2}\theta +\zeta_{⊥}\sin^{2}\theta }{\sin\theta }\right)$$

(11) $$B=2\pi n{\sigma^{\prime }}^{2}\left(\zeta_{\left|\right|}-\zeta_{⊥}\right)\cos\theta$$

(12) $$C=2\pi n{\sigma^{\prime }}^{3}\left(\frac{\zeta_{⊥}\cos^{2}\theta +\zeta_{\left|\right|}\sin^{2}\theta }{\sin\theta }\right)$$

where n is the number of helical turns, $\sigma^{\prime }$ is the average radius of the helix (m), and θ is the helix angle residual (°). The tangential drag coefficient $\zeta_{\left|\right|}$ and normal drag coefficient $\zeta_{⊥}$ are the viscous drag coefficients of the arc segments in the liquid, and for the elongated helical structure, Lighthill proposed the drag coefficient equation to be [34]:

(13) $$\zeta_{\left|\right|}=\frac{2\pi \eta }{\ln\left(\frac{0.36\pi \sigma^{\prime }}{r\sin\theta }\right)}\zeta_{⊥}=\frac{4\pi \eta }{\ln\left(\frac{0.36\pi \sigma^{\prime }}{r\sin\theta }\right)+1/2}$$

where η is the dynamic viscosity of the fluid (Pa·s), λ is the helical pitch (m), and r is the helical cross-section radius (m), and for the structure of this paper, its equivalent radius can be expressed as $r=\sqrt{ab\pi /\pi }=\sqrt{ab}$. The bottom surface of the tail-carrying cavity has a certain degree of taper, which is approximated to be a cylinder in order to facilitate the calculations, with a diameter of d and a height of h. In a low-Reynolds number environment, the viscous drag force on a cylinder moving in its axial direction is related to the velocity of the cylinder motion u, the equivalent cross-sectional area of the cylinder A, the fluid density ρ, and the drag coefficient $C_{d}$, which can be expressed as:

(14) $$F_{d}=\frac{1}{2}\rho C_{d}Au^{2}.$$

For a moving object in a liquid with Re≤1, the drag coefficient is Cd=24/Re, and the equivalent cross-sectional area A of the cylinder can be calculated from the volume method:

(15) $$\frac{4}{3}\pi {\left(\frac{D}{2}\right)}^{3}=\pi {\left(\frac{d}{2}\right)}^{2}h^{\prime }$$

(16) $$A=\pi \frac{D^{2}}{4}$$

where D is the equivalent diameter of the cylinder (mm). From (14)–(16), the magnitude of viscous drag force experienced is:

(17) $$F_{d}=3\pi \eta Du.$$

Then, the translational drag coefficient of the drug-carrying cavity portion can be expressed as:

(18) $$\psi_{u}=3\pi \eta D.$$

For the cylinder in a low-Reynolds number environment, the rotational drag coefficient can be expressed as:

(19) $$\psi_{\omega }=\pi \eta h^{\prime }d^{2}.$$

Combining the front helical structure of the microrobot with the rear drug-carrying cavity, the translational drag coefficient and the rotational drag coefficient of the drug-carrying cavity are introduced in the matrix of helical propulsion:

(20) $$\left[\begin{array}{c} F\\ \tau \end{array}\right]=\left[\begin{array}{cc} A+\psi_{u} & B\\ B & C+\psi_{\omega } \end{array}\right]\left[\begin{array}{c} u\\ \omega \end{array}\right].$$

Performing the transformation yields:

(21) $$\left[\begin{array}{c} u\\ \tau \end{array}\right]=\left[\begin{array}{cc} \frac{1}{A+\psi_{u}} & \frac{-B}{A+\psi_{u}}\\ \frac{B}{A+\psi_{u}} & C+\psi_{\omega }-\frac{-B^{2}}{A+\psi_{u}} \end{array}\right]\left[\begin{array}{c} F\\ \omega \end{array}\right].$$

Then, the motion velocity u of the microrobot can be expressed as:

(22) $$u=\frac{F}{A+\psi_{u}}-\frac{\omega B}{A+\psi_{u}}.$$

Substituting into (10)–(12) gives:

(23) $$u=\frac{\sin\theta \left(F-2\pi \omega n{\sigma^{\prime }}^{2}(\zeta_{\left|\right|}-\zeta_{⊥})\cos\theta \right)}{2\pi n\sigma^{\prime }\left(\zeta_{\left|\right|}\cos^{2}\theta +\zeta_{⊥}\sin^{2}\theta \right)+3\pi \eta D\sin\theta }.$$

When only a rotating uniform magnetic field is applied and the wandering state of the magnetically driven microrobot reaches stability, the combined force *F* of the power externally applied to the microrobot and the resistance acting on the microrobot is zero, so the wandering speed of the microrobot can be calculated according to the following equation:

(24) $$u=\frac{-2\pi \omega n{\sigma^{\prime }}^{2}(\zeta_{\left|\right|}-\zeta_{⊥})\sin\theta \cos\theta }{2\pi n\sigma^{\prime }\left(\zeta_{\left|\right|}\cos^{2}\theta +\zeta_{⊥}\sin^{2}\theta \right)+3\pi \eta D\sin\theta }.$$

According to the motion in the low-Reynolds number fluid environment, the tangential and normal forces in the helix part can be expressed by the resistance theory as:

(25) $$\begin{array}{l} F_{s}=f_{s}=-n\lambda \zeta_{\left|\right|}\omega \sigma^{\prime }\sin\theta \\ F_{n}=f_{n}=-n\lambda \zeta_{⊥}\omega \sigma^{\prime }\cos\theta \end{array}.$$

Then, the driving force generated in the forward direction of the microrobot during the uniform rotation forward can be expressed as:

(26) $$\begin{array}{l} F_{z}=F_{s}\cos\theta -F_{n}\sin\theta \\ =n\lambda \omega \sigma^{\prime }\sin\theta \left(\zeta_{⊥}-\zeta_{\left|\right|}\right) \end{array}.$$

The torque required by the microrobot is:

(27) $$\begin{array}{l} L_{z}=-\sigma^{\prime }\left(F_{s}\sin\theta +F_{n}\cos\theta \right)\\ =n\lambda \omega {\sigma^{\prime }}^{2}\frac{\left(\zeta_{⊥}\cos^{2}\theta +\zeta_{\left|\right|}\sin^{2}\theta \right)}{\cos\theta } \end{array}.$$

## 3. Preparation Method

**Figure 2:**
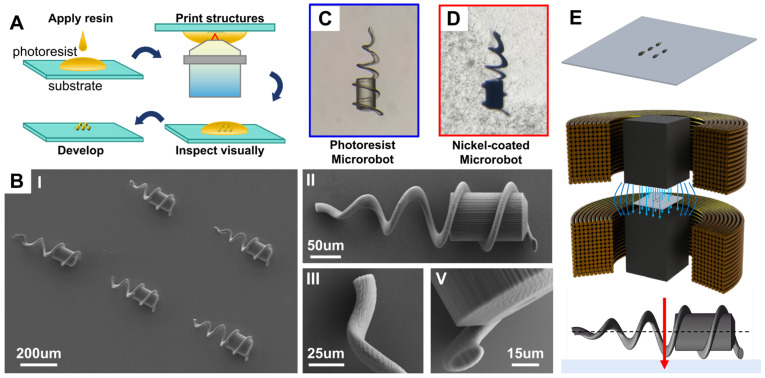
Microrobot preparation. (**A**) Two-photon femtosecond laser printing process. (**B**) Microrobot SEM scanning test image. (**C**) Microrobot without nickel plating layer. (**D**) Microrobot with nickel plating layer. (**E**) Magnetization along short axis of robot.

The femtosecond laser two-photon polymerization 3D printing technology used in this paper for the preparation of the microrobot body is based on the Photonic Professional (GT) two-photon 3D printer produced by Nanoscribe, Eggenstein-Leopoldshafen, Germany, and the printing material used is IP-Dip photoresist, developed by Nanoscribe. The photoresist is a negative photoresist, and during the lithography process, the IP-Dip photoresist is coated on the substrate surface and locally polymerized at the desired location using a femtosecond laser to form the desired structure and pattern, and then the unpolymerized portion is removed using a developer solution to leave behind the polymerized structure, as shown in Figure 2C. The microrobot structure prepared using this technique is not magnetic, so the printed microrobot needs to be sputtered with magnetic particles using magnetron sputtering to attach a 200 nm thick nickel metal layer to its surface. Figure 2D shows the image of the structure observed under a microscope after magnetron sputtering—the surface is obviously darkened and a nickel metal layer is deposited on the surface. Finally, according to the motion control mechanism of the microrobot, magnetization was performed along the direction of the short axis of the microrobot to improve the efficiency of helical propulsion. The method of magnetization along the short axis of the microrobot is as follows: using two electromagnet coils arranged symmetrically up and down, a substrate containing several magnetron-sputtered microrobots is placed between two electromagnet cores, with the core axis perpendicular to the substrate. Then, the two electromagnet coils are fed with a current in the same direction, which generates a magnetic field strength of more than 1.5T. Since the microrobots lie flat on the substrate, the magnetization is carried out in such a way that the direction of magnetization is exactly in the direction of the short axis of the microrobots.

## 4. Experimental Study of Motion Characteristics

**Figure 3:**
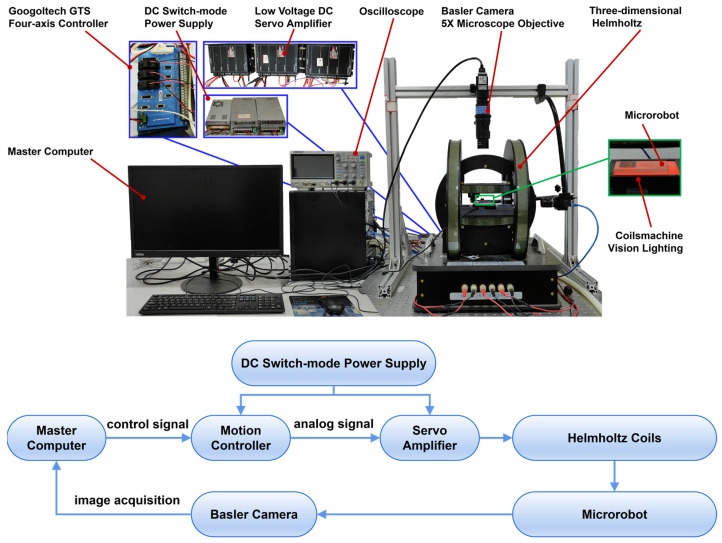
Three-axis Helmholtz coil magnetic field-driven stage.

In order to verify the motion performance of the magnetically controlled microrobot, our team built a three-axis Helmholtz coil magnetic field drive platform by adjusting the input currents of electromagnetic coils in different directions, so as to form a uniform rotating magnetic field in the center field space; the experimental system is shown in Figure 3. The system adopts a multi-threaded architecture, including the main control thread, the coil drive thread, and the visual feedback thread—the main control thread consists of the master computer and the motion controller (Gugo GTS 4-axis controller, Googoltech, Shenzhen, China); the coil drive thread consists of three pairs of low-voltage DC servo amplifiers, a power supply, and three sets of Helmholtz coils; and the visual feedback thread consists of two basler cameras and 5X microscope objectives, as well as a conilsmachine vision lighting. The master computer is electrically connected to a motion controller, the motion controller is electrically connected to a servo amplifier, the servo amplifier is electrically connected to the Helmholtz coils, the Helmholtz coils are magnetically connected to the microrobot, there is a binocular orthogonal camera set up outside the Helmholtz coils, and the binocular orthogonal camera signals are connected to the receiving end of the master computer, which forms a closed-loop control. The control system mainly consists of two parts: the magnetic field drive control system and the image processing system. The magnetic field drive control system is responsible for the generation of Helmholtz coil drive signals and the generation of motion control algorithms; the image processing system includes the real-time acquisition of images and the detection of the microrobot, which feeds the position information of the microrobot to the host computer and assists in the completion of the microrobot motion control algorithm.

According to the previous analysis, it can be seen that, when driven by the rotating magnetic field, the magnetically controlled micro–nano soft robot will be subjected to the action of the magnetic torque, so as to rotate around its short axis, and the helical structure of the microrobot interacts with the liquid in the process of rotating to generate propulsion, which in turn drives the microrobot forward. In order to fully verify the swimming performance of the robot, this paper prepares the microrobot with different structural dimensions, and the structural parameters are shown in Table 1.

**Figure 4:**
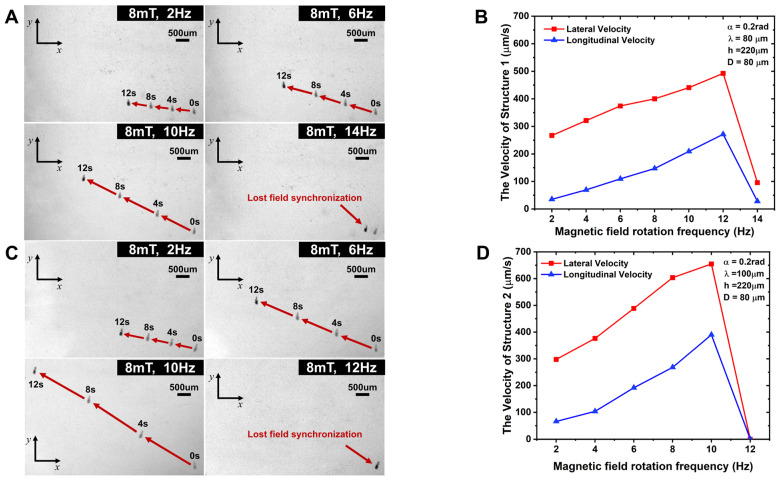
Motion path diagram and motion velocity curves of No. 1,2 microrobots. (**A**) Motion path diagram of No. 1 microrobot at 2 Hz–14 Hz rotational frequency. (**B**) Longitudinal/lateral motion velocity profile of No. 1 microrobot. (**C**) Motion path diagram of No. 2 microrobot at 2 Hz–12 Hz rotational frequency. (**D**) Longitudinal/lateral motion velocity profile of No. 2 microrobot.

**Figure 5:**
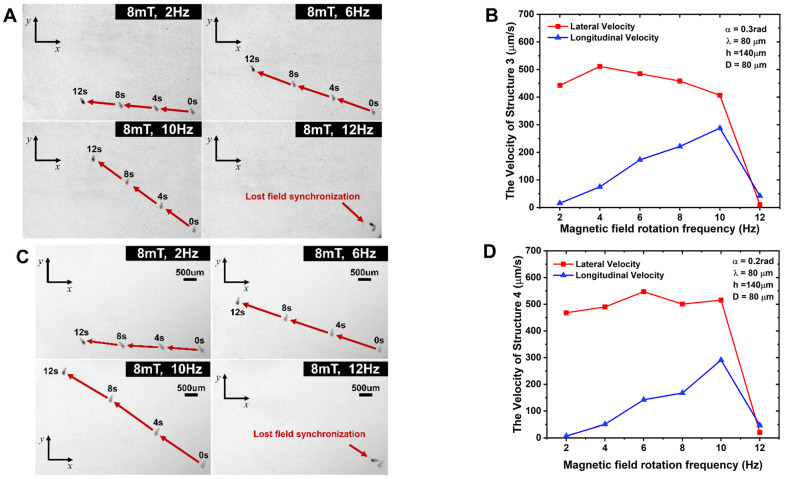
Motion path diagram and motion velocity curves of No. 3,4 microrobots. (**A**) Motion path diagram of No. 3 microrobot at 2 Hz–12 Hz rotational frequency. (**B**) Longitudinal/lateral motion velocity profile of No. 3 microrobot. (**C**) Motion path diagram of No. 4 microrobot at 2 Hz–12 Hz rotational frequency. (**D**) Longitudinal/lateral motion velocity profile of No. 4 microrobot.

**Figure 6:**
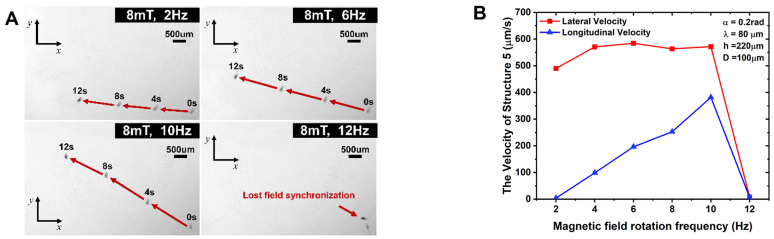
Motion path diagram and motion velocity curves of No.5 microrobot. (**A**) Motion path diagram of No.5 microrobot at 2 Hz–12 Hz rotational frequency. (**B**) Longitudinal/lateral motion velocity profile of No.5 microrobot.

**Figure 7:**
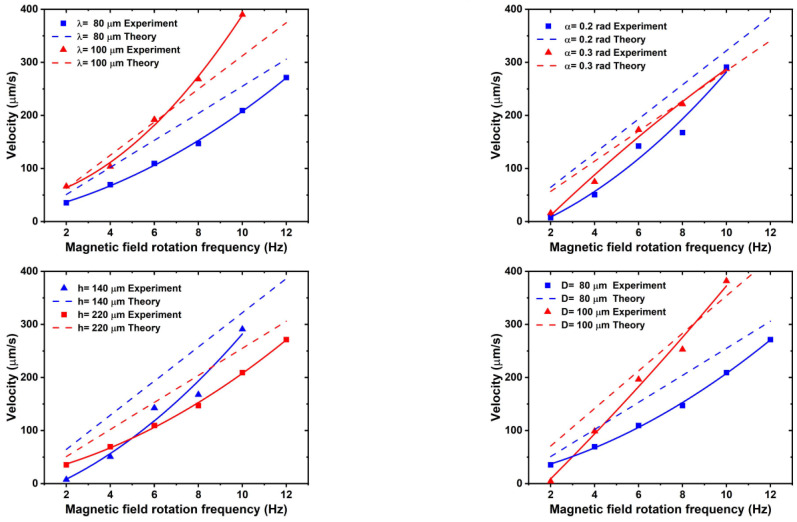
Experimental and theoretical comparison curves of longitudinal velocities of microrobots.

The experiment applies a rotating magnetic field strength with a frequency of 2–14 Hz. A CCD camera is used to take images and observe the motion state of the microrobot. During the experiment, the frequency of the rotating magnetic field is gradually increased at intervals of 2 Hz, and the position of the microrobot is recorded at intervals of every 4 s. Through the test, the motion position and attitude of the microrobot were analyzed and the motion velocity was calculated, and the microrobot velocity–frequency change curve was plotted, as shown in Figure 4, Figure 5, Figure 6 and Figure 7.

Figure 4 shows the motion paths and longitudinal and lateral motion curves of the microrobots with different helical pitch sizes, and the overall analysis shows that the longitudinal and lateral motion velocities of the microrobots have similar trends, which increase with increases in the rotating magnetic field frequency, but the microrobots with larger pitches have higher motion velocities. The critical out-of-step frequencies of the No. 1 and No. 2 microrobots are 14 Hz and 12 Hz, respectively. When the out-of-step frequency is greater than that, the robot stops moving forward and remains stationary. After the out-of-step frequency, the robot stops moving forward and remains stationary. At this point, the spiral propulsion ability of the microrobot cannot follow the external rotating magnetic field changes, resulting in the out-of-step frequency [35]. As can be seen from the motion path of the microrobot in Figure 4A,C, due to the asymmetric characteristics of the helix coupled with the friction generated by the bottom surface, the microrobot is propelled forward while accompanied by lateral motion. With increases in the rotating magnetic field frequency and the helical pitch, the speed of the lateral motion increases. For example, the microrobot (No. 1) with a helical pitch of 80 µm has a lateral velocity of 492.35 µm/s and a longitudinal velocity of 271.32 µm/s at a rotating magnetic field frequency of 12 Hz, while the microrobot (No. 2) with a helical pitch of 100 µm has a lateral velocity of 654.7 µm/s and a longitudinal velocity of 390 µm/s at a frequency of 10 Hz. The ratio of lateral motion velocity to longitudinal motion velocity at all frequencies (less than 12 Hz) for microrobot No. 1 is greater than the lateral-to-longitudinal ratio for microrobot No. 2.

Figure 5 shows the motion paths and longitudinal and transverse motion curves of the microrobots with different helical cone angles. From the figure, it can be seen that the longitudinal motion velocity increases with increases in the magnetic field frequency, while the lateral motion velocity is relatively less affected by the rotation frequency of the magnetic field, and the corresponding out-of-step frequencies are all about 12 Hz. By comparative analysis, we can see that the motion velocity of the microrobot decreases with the increase in the helical cone angle. For example, the lateral motion velocity of the microrobot (No. 4) with a cone angle of 0.2 rad reaches 515.35 µm/s at 10 Hz, and its longitudinal motion velocity reaches 290.87 µm/s, whereas the lateral motion velocity of the microrobot (No. 3) with a cone angle of 0.3 rad reaches 406.8 µm/s at 10 Hz, and its longitudinal motion velocity reaches 287.7 µm/s. However, comparing the lateral and longitudinal velocities of the two at each frequency, the ratio of the lateral and longitudinal velocities of microrobot No. 4 is greater than that of microrobot No. 3, i.e., the microrobot with a cone angle of 0.3 rad has a more stabilized spinning motion.

Figure 4A,B and Figure 6 show the motion path diagrams with longitudinal and lateral motion curves of the microrobots with different cavity diameters. As can be seen from the figures, the longitudinal motion speeds of the two structures also increase with increases in the rotating magnetic field frequency. Comparative analysis shows that the highest longitudinal velocities of the two structures, No. 5 and No. 1, are as follows: the longitudinal velocity of the microrobot with a cavity diameter of 80 µm (No. 1) is 271.32 µm/s; and the longitudinal velocity of the microrobot with a cavity diameter of 100 µm (No. 5) is 381.81 µm/s. Therefore, the longitudinal motion velocity increases with increases in the cavity diameter. The ratio of the lateral to longitudinal velocities of microrobot No. 5 is generally smaller than that of microrobot No. 1 at all frequencies.

From Figure 4, Figure 5 and Figure 6, we can see that the ratio of the lateral motion speed to the longitudinal motion speed of the microrobot decreases with increasing frequency. The lateral-to-longitudinal ratio of microrobot No. 1 is 2.108 at a frequency of 10 Hz, and that of microrobot No. 2 is 1.679 at a frequency of 10 Hz. The lateral-to-longitudinal ratio of microrobot No. 3 is 1.772 at a frequency of 10 Hz, and that of microrobot No. 4 is 1.414 at a frequency of 10 Hz. The lateral-to-longitudinal ratio of microrobot No. 5 is 1.497 at a frequency of 10 Hz.

As can be seen in the above analysis, the motion speed of the helical microrobot is mainly affected by the frequency of the rotating magnetic field and the structural size, and under the same magnetic field strength, the longitudinal speed increases with increasing frequency, but the lateral offset increases as well. In addition, at the same frequency, as the helical pitch and cavity diameter increase and the helical cone angle decreases, the longitudinal and lateral velocities of the microrobot increase. While analyzing the ratios of lateral and longitudinal motion velocities for the microrobots of each structure, it could be seen that the lateral and longitudinal velocities of the microrobots with a larger pitch, larger helical cone angle, and larger cavity diameter are smaller. Therefore, considering the longitudinal velocity and the lateral offset, while considering each structural parameter in order to obtain a larger velocity, it is necessary to combine the consideration of the unfavorable effects of larger ratios of lateral and longitudinal velocities in order to obtain the optimal size.

In order to validate and revise the theoretical model of the microrobot, the first established microrobot velocity model was fitted and the model parameters were optimized to minimize the residuals between the model’s predicted values and the actual observed values. A set of parameter values—k_1_, k_2_, k_3_—were obtained using appropriate statistical methods and minimum objective optimization algorithms, enabling the model to more accurately describe the trend of the actual data, as follows:

(28) $$u=\frac{k_{1}\cdot (-2\pi \omega n{\sigma^{\prime }}^{2}(\zeta_{\left|\right|}-\zeta_{⊥})\sin\theta \cos\theta )}{k_{2}\cdot 2\pi n\sigma^{\prime }\left(\zeta_{\left|\right|}\cos^{2}\theta +\zeta_{⊥}\sin^{2}\theta \right)+k_{3}\cdot 3\pi \eta D\sin\theta }$$

where k_1_ = 1.2, k_2_ = 0.6, k_3_ = 1.0.

Figure 7 shows that the longitudinal motion speeds of the microrobots, split into five groups according structural size in terms of cone angle, helical pitch, helical length, and cavity diameter, have been compared with the theoretical motion speeds. Overall analysis shows that the longitudinal and lateral motion velocities of the microrobots increase with increases in the rotating magnetic field frequency (within the out-of-step frequency), and the microrobots with a larger cavity diameter and larger helical pitch exhibit a faster spinning speed, while the microrobots with a smaller helix height and smaller helix cone angle outperform the other structures with the same feature sizes.

As shown in Figure 7, the kinematic characteristic curves are basically consistent with the theoretical velocity model, indicating that the model can accurately predict the helical propulsion velocity of the microrobots. However, it can be observed that the velocity profile of the microrobot in the experiment does not show the linear relationship expected from the theory. There may be certain disturbing factors such as vibration and boundary effects during the experiment. According to the analysis, due to the enhanced viscous effect of the fluid in the low-Reynolds number environment, the flow is more susceptible to microscopic inhomogeneities, resulting in velocity fluctuations due to non-uniform influences on the experimental structure [36].

## 5. Conclusions

In this paper, a magnetically controlled cone-helix soft microrobot structure with a drug-carrying function is proposed; its magnetically controlled mechanism, kinetic model, and microrobot preparation have been analyzed; and control experiments of the microrobot have been performed. The paper’s main contents are as follows:

1. A magnetically controlled cone-helix soft microrobot structure is proposed, which consists of a conical helix part with an elliptical cross-section, and the helix has a certain taper angle with a drug-carrying cavity at the tail. A mathematical model of the magnetically controlled microrobot in a low-Reynolds number fluid environment was established based on the resistance theory and slender body theory, and the magnetically controlled microrobot was also prepared based on two-photon femtosecond laser 3D printing and magnetron sputtering technology.
2. Under the conditions of 8 mT magnetic field strength and 2–14 Hz magnetic field rotation frequency, motion control experiments are carried out on the magnetically controlled cone-helix soft microrobot, and the propulsion characteristics of the microrobot are analyzed. The results show that the microrobot structure proposed in this paper can be stabilized for helical propulsion under the control of the rotating magnetic field; the overall motion speed is affected by the rotation frequency and the structure size; and the longitudinal speed increases with increases in frequency under the same magnetic field strength, but the lateral offset also increases in the same way. In addition, at the same frequency, with increases in helical pitch and cavity diameter as well as decreases in the helical cone angle, the longitudinal and lateral speeds of the microrobot increase. The lateral and longitudinal velocities of the microrobot with a larger pitch, larger helical cone angle, and larger cavity diameter are relatively small. When designing the dimensions, the longitudinal velocity and lateral offset velocity need to be considered comprehensively to obtain the optimal structural dimensions. The magnetically controlled cone-helix soft microrobot proposed in this paper is able to achieve a spin-in velocity of more than 1 body length/s in a rotating magnetic field of 8 mT. For example, the microrobot (No. 2) with a cone angle of 0.2 rad, a helical pitch of 100 µm, a helical height of 220 µm, and a cavity diameter of 80 µm can achieve a longitudinal spin-in motion speed of 1.08 body length/s, or 390 µm/s.

The microrobots prepared in this report have only been sputtered with a nickel film and are not biocompatible. Our group plans to further extend these research experiments in future work by sputtering a metallic titanium film on the surface of the microrobot on top of the sputtered nickel film to make the microrobot biocompatible [37]. In addition, efforts will be made to further improve the motion rate and motion stability of the microrobot, and at the same time, the functionality of the microrobot in loading and releasing drugs will be explored and experimentally analyzed, so as to further provide a theoretical basis for the microrobot proposed in this paper to be applied in the actual medical field. Moreover, better experimental methods are sought in order to conduct experiments with smaller-sized microrobots, so that the microrobots do not cause problems such as blood vessel blockage in the blood or body fluid circulatory system. Further advancing the exploration of cluster control of microrobots would also aid in efficient treatment. In addition, the team is highly interested in finding a solution to the issues with microrobot recycling and degradation, which is a major direction for the team in future work, and when this problem is solved, the impacts of microrobots on the human body and the environment can be reduced.

## Figures and Tables

**Table 1:** Dimensional parameters of robots with different structural dimensions.

No.		1	2	3	4	5
Cone angle	α/rad	0.2	0.2	0.3	0.2	0.2
Helical pitch	λ/μm	80	100	80	80	80
Helical height	h/μm	220	220	140	140	220
Cavity diameter	D/μm	80	80	80	80	100
Cavity wall thickness	δ/μm	8	8	8	8	8
Cavity caliber	d/μm	6	6	6	6	6
Cavity height	h/μm	120	120	120	120	120
Body length	L/μm	360	360	280	280	360

## Data Availability

The original contributions presented in the study are included in the article, further inquiries can be directed to the corresponding authors.
